# Quantification of ^99m^Tc-DPD concentration in the lumbar spine with SPECT/CT

**DOI:** 10.1186/2191-219X-3-45

**Published:** 2013-06-05

**Authors:** Michal Cachovan, Alexander Hans Vija, Joachim Hornegger, Torsten Kuwert

**Affiliations:** 1 Pattern Recognition Laboratory, FAU Erlangen-Nuremberg, Martensstrasse 3, Erlangen 91058, Germany; 2 Erlangen Graduate School in Advanced Optical Technologies, Erlangen 91058, Germany; 3 Clinic of Nuclear Medicine, FAU Erlangen-Nuremberg, Ulmenweg 18, Erlangen 91054, Germany; 4 Siemens Healthcare, Molecular Imaging, 2501 North Barrington Road, Hoffman Estates 60192-2061 IL, USA

**Keywords:** Quantitative SPECT, SPECT/CT, Bone scintigraphy

## Abstract

**Background:**

Routine single-photon emission computed tomography (SPECT) currently lacks quantitative information on regional activity concentration (ACC) of the injected tracer (e.g. kBq/ml). Furthermore, little is known on the skeletal absolute concentration of ^99m^Tc-DPD after intravenous injection in bone scintigraphy. The aim of this study is to determine ACC in the healthy lumbar vertebrae of patients using a recently published quantitative SPECT/computed tomography (CT) protocol.

**Methods:**

Lumbar vertebrae ACC estimates were performed in 50 female patients (mean age 69.88 ± 13.73 years) who had been administered 562.84 ± 102.33 MBq of ^99m^Tc-DPD and had undergone SPECT acquisition 4 h after the injection. The SPECT/CT system was calibrated against a well counter. Images were reconstructed with Flash3D. ACC and CT tissue density were measured in volumes of interest drawn over the spongious bone tissue of the three lower lumbar vertebral bodies when these exhibited no focal CT or SPECT pathology.

**Results:**

Average ACC measured in the normal spongious bone tissue was 48.15 ± 13.66 kBq/ml (95% confidence interval (CI) 45.81 to 50.50 kBq/ml). This corresponds to a mean standardised uptake value (SUV) of (5.91 ± 1.54) (95% CI (5.64 to 6.17) SUV). SUV correlated significantly with Hounsfield units (HU) (*r* = 0.678, *p* < 0.0001). Significant negative correlations were observed between age and HU (*r* = −0.650, *p* < 0.0001) and between age and SUV (*r* = −0.385, *p* < 0.0001).

**Conclusions:**

The SUVs determined for ^99m^Tc-DPD uptake 4 h post injection are in the same range as those reported for [^18^F]fluoride in positron emission tomography. The strong correlation of SUV with bone CT density underlines the physiological significance of this variable. Our data suggest further investigation of the potential value of ACC measurement in the diagnosis of pathological conditions such as osteoporosis or in following up osseous metastases under therapy.

## Background

Quantitative single-photon emission computed tomography (QSPECT) was first introduced into nuclear medicine in the 1990s. Rosenthal et al. predicted in 1995 that estimates of absolute SPECT tracer concentration would enter the clinical arena in the near future [1]. However, to date, their prediction has not come true as only few approaches to QSPECT have been established in clinical practice (for reviews, see Ritt et al. [2] and Bailey et al. [3]).

Gilland et al. [4] and Tsui et al. [5,6] have shown that several physical phenomena have to be taken into account to achieve absolute quantification in SPECT. One of the first QSPECT protocols validated in humans was reported by Willowson et al. [7] who quantified the concentration of ^99m^Tc in the cardiac cavity of patients studied by radionuclide ventriculography. Also our research group [8] proposed a QSPECT protocol to quantify the tissue concentration of ^99m^Tc. Our methodology, as well as that by Willowson et al., involves the use of co-registered data from X-ray computed tomography (CT) to correct for attenuation and a window subtraction approach (dual energy window) to correct for scattered counts.

In a recent review paper, Bailey and Willowson [3] predict a strong research interest in quantitative SPECT in the near future and suggest quantitative bone ^99m^Tc scanning to be one of the potential fields of clinical interest. However, little is known on the accumulation of bone-seeking SPECT radiopharmaceuticals in the skeleton in absolute terms. Before the advent of hybrid imaging combining SPECT with CT, Israel et al. [9] used an empirical method to quantify osseous concentration of ^99m^Tc-methylene diphosphonate (MDP). Their approach did not include corrections for attenuation. Therefore, the standard deviations in their results of lumbar tracer concentration amounting 30% to 40% of the mean were inevitable, motivating further research in that field.

Building on the methodological set-up developed by Zeintl et al. [8], we measure in this study the activity concentration (ACC) of ^99m^Tc-diphosphono-propanedicarboxylic acid (DPD) in the spongious bone tissue without focal SPECT and CT abnormalities in women referred for bone scintigraphy. Furthermore, we correlate tracer concentration with bone density and age. Due to the dependence of Hounsfield units (HU) on various factors including patient shape and size [10], we also compute and collect linear attenuation coefficients (LAC) [11] and investigate their correlation with ACC.

## Methods

In order to obtain quantitative results, multiple calibrations were necessary. Calibrations were performed using a protocol proposed by Zeintl et al. [8]. We adopt and cite the proposed methods in the following description.

### Patients

*In vivo* patient data analysis was carried out with permission from the Ethical Committee of the University of Erlangen-Nuremberg. We acquired data for a group of 50 female patients (mean age 69.88 ± 13.73 years; minimum 25 years, maximum 97 years) undergoing ^99m^Tc-DPD bone scintigraphy of the lumbar region between February and August 2012 using a standard SPECT/CT protocol as described in the study of Zeintl et al. [8]. The injected activity of ^99m^Tc ranged from 5.60 to 10.30 MBq/kg (mean 8.26 ± 1.41 MBq/kg) corresponding to 151 to 278 μCi/kg (mean 223 ± 38 μCi/kg). The mean total activity administered was equal to 562.84 ± 102.33 MBq (15.21 ± 2.77 mCi). SPECT/CT was performed 4 h after intravenous injection.

The patients in our group were included based on the following criteria:

• Start of SPECT acquisition within an interval of 4 h ± 30 min post injection

• Access to data on measured injection activity, time of measurement, time of injection, residual activity measurement and time of residual activity measurement

• Access to patient's weight and height information

• At least one vertebral body of the lumbar vertebrae (LV 3, 4 and 5) in the field of view (FOV) of both SPECT and CT acquisitions

• Absence of any kind of metal or surgical implants in the LV FOV

Based on patient records, there are two main groups of patients: those studied for work up of back pain (22) and those examined for staging malignancy (28). In 22 cases of the latter, the primary malignancy had been diagnosed in the breast. One woman was diagnosed with both breast and ovarian cancer. There was one case involving each of the following carcinomas: ovarian, oesophageal, renal cell and Ewing's sarcoma and osteoid osteoma.

All three LVs in the 45 patients examined and only two LVs in the 5 patients examined were in the FOV. Overall, 145 LVs were considered in this study.

### Data acquisition and reconstruction

We used both Symbia T6 and Symbia T2 (CT with a maximum of six and two slice acquisitions per rotation, respectively, Siemens Healthcare, Molecular Imaging, Hoffman Estates, IL, USA) system at the Nuclear Medicine Clinic in Erlangen, Germany to acquire *in vivo* patient data. SPECT scans were acquired using low-energy high-resolution collimation, a 128 × 128 matrix of 4.8-mm pixel size and a total of 120 projections over 360° with a dwell time of 15 s/view. Subsequent to the SPECT acquisition, a low-dose CT scan was acquired with 130 kV and 30 ref mAs using adaptive dose modulation (CARE Dose 4D available for both Symbia® T6 and T2; Siemens Healthcare [12]). The CT data were generated with a 2.5-mm slice thickness using a smooth reconstruction kernel (B08s, Siemens Healthcare [8]) and a 1-mm slice thickness using a medium kernel (B41s, Siemens Healthcare).

SPECT reconstruction was performed using Flash3D (Siemens Healthcare, Molecular Imaging) [13,14]. Flash3D is an ordered subset expectation maximisation (OSEM) reconstruction algorithm with depth-dependant 3D (axial and trans-axial) resolution recovery, scatter correction using scatter window subtraction (dual energy window approach) and attenuation correction based on attenuation maps derived from the CT data filtered with the B08s kernel. The OSEM SPECT reconstruction used four subsets and eight iterations without post-smoothing, as suggested by Zeintl et al. [8].

### Data analysis

From the 145 vertebral bodies (LV 3 to 5), all vertebrae exhibiting any focal SPECT or CT pathology in their spongious bone tissue were excluded from the analysis based on the diagnosis defined by an experienced physician. The rationale for this was the intention to measure ACC in spongious bone tissue not affected by malignancy, compression fractures, osteochondrosis or facet disease. Overall, 133 vertebrae were selected for analysis based on the criterion previously defined. Five LV 5, three LV 4 and four LV 3 were rejected.

**Figure 1:**
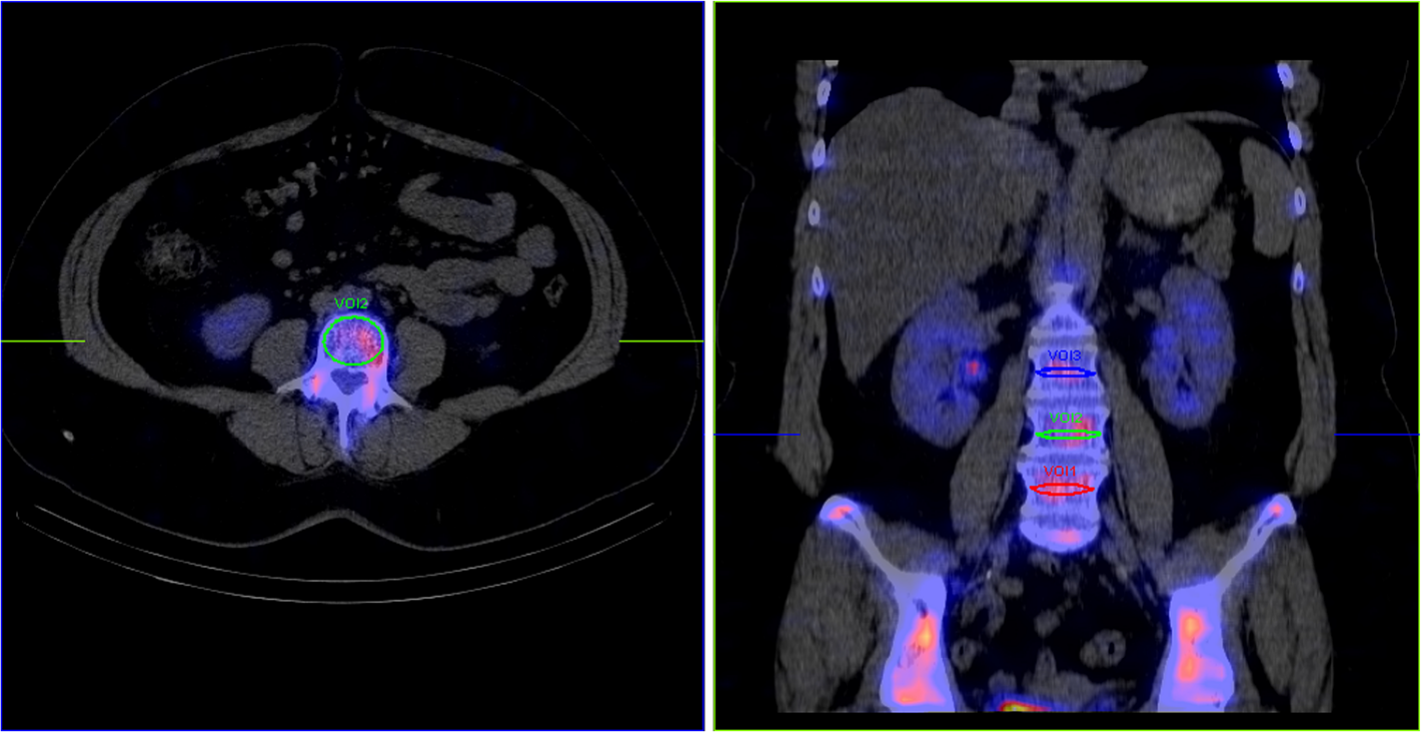
**Transverse and coronal images of a patient's SPECT/CT fused data sets including the three lower LV.** The ellipses depict the VOIs selected.

The delineation of the volumes of interest (VOIs) was performed by an experienced physician using the regular clinical volumetric analysis tool provided by the camera's vendor (e.soft, Volumetric Analysis, v 1.1.6.1, Siemens Healthcare, Molecular Imaging) which reports the statistics for the various measures (HU and counts). The B41s kernel filtered CT data were used to derive the VOI selection in a volumetric fusion display. Elliptical VOIs were hand-drawn in the lower three lumbar vertebrae such that the borders coincided with the vertebral cortical bone shell. The average diameter for the selected disc was 31.81 ± 2.08 mm. We adjusted the axial size of the VOI in order to delineate a volume of approximately 2 ml. Figure 1 shows a representative patient's fused data set in transverse and coronal planes with their respective VOIs. The mean volume of all the selected VOIs was 2.06 ± 0.14 ml. For comparison, we also selected a VOI which covered the complete vertebral body, resulting in an average volume of 10.63 ± 2.20 ml with the same average diameter in the selected disc. We recorded the overall reconstructed counts, the mean HU, the mean LAC and the size of the VOI for later analysis. We calculated absolute activity concentration in accordance with the work of Israel et al. [9]. First, a calibration of the SPECT/CT system with a uniform phantom is performed which provides a volume sensitivity factor and is specific to the camera type, collimator type and the window energy settings used. The patient's reconstructed values are then normalised with volume sensitivity, allowing the calculation of ACC. All data were decay-corrected to the time of injection in order to control fluctuations at the start time of the acquisition. The administered activity was corrected for residual activity in the syringe. Final values of quantitative tracer concentrations are thus defined with respect to injection time. Body weight (BW), lean body weight (LBW) and body surface area (BSA) computed based on other works [15,16] were used to study standardised uptake value (SUV) normalisation variations, namely SUV_BW_ (Equation 1), SUV_LBW_ (Equation 2) and SUV_BSA_ (Equation 3).

(1) $$\mathrm{SU}V_{\mathrm{BW}}=\frac{\mathrm{ACC}\times \text{WEIGHT}}{\text{INJECTED ACTIVITY}}$$

(2) $$\mathrm{SU}V_{\mathrm{LBW}}=\frac{\mathrm{ACC}\times \left(1.07\times \text{WEIGHT}\right)\times 148\times \frac{{\text{WEIGHT}}^{2}}{{\text{HEIGHT}}^{2}}}{\mathrm{INJECTED ACTIVITY}}$$

(3) $$\mathrm{SU}V_{\mathrm{BSA}}=\frac{\mathrm{ACC}\times {\mathrm{HEIGHT}}^{0.725}\times {\mathrm{WEIGHT}}^{0.425}\times 0.007184}{\mathrm{INJECTED ACTIVITY}}$$

We also computed, based on the work of Zeintl et al. [8], the ACC underestimation for the selected VOIs. This was accomplished using the emission recovery coefficient tables for our particular operation point (VOI size and acquisition parameters) as defined in the work of Zeintl et al. [8]. These recovery coefficients were obtained using simulations of hot spheres of different diameters in hot bath and were validated using real phantom acquisitions.

### Statistical analysis

In the ‘Results’ subsection, we report various quantitative measures, including bone density expressed in HU and tracer ACC expressed in kilobecquerel per millilitre (kBq/ml) and SUV. The data are described by their mean values, their standard deviations and their 95% confidence intervals (CI). The correlation of the variables is analysed using linear regression yielding Pearson's correlation coefficients. The statistical significance of differences in the correlation coefficients was tested using Fisher's transformation (*z* and *p* values). Significance was accepted at *p* < 0.05. Statistical analysis was performed using MedCalc for Windows, version 11.5.0.0 (MedCalc Software, Mariakerke, Belgium).

## Results and discussion

### Results

Table 1 shows the statistical summary for the various measures collected. Mean vertebral VOI ACC for the patient group was 48.15 ± 13.66 kBq/ml (95% CI, 45.81 to 50.50 kBq/ml) or 5.91 ± 1.54 SUV_BW_ (95% CI, 5.64 to 6.17 SUV_BW_). The VOI analysis showed a mean CT value of 106.52 ± 58.00 HU (95% CI, 96.57 to 116.46 HU) and a mean attenuation coefficient of 0.168 ± 0.007 cm^−1^ (95% CI, 0.167 to 0.170 cm^−1^). The mean ^99m^Tc-labelled tracer uptake percentage was in the range of 8.75 × 10^−3^ ± 2.23 × 10^−3^ (95% CI, 8.12 to 9.39 × 10^−3^) injected dose (% ID) per millilitre of vertebral spongious bone.

**Table 1:** Statistical analysis of all variables collected for 50 female patients and 133 vertebral bodies

**Variable**	**Mean**	**SD**	**95% CI**
Age (years)	69.88	13.73	65.98 to 73.78
Weight (kg)	69.39	13.38	65.59 to 73.19
Height (cm)	160.72	6.05	159.00 to 162.44
Administered activity (MBq)	562.84	102.33	533.76 to 591.92
Activity per 1 kg BW (MBq/kg)	8.26	1.41	7.86 to 8.66
ACC (kBq/ml)	48.15	13.66	45.81 to 50.50
ACC (SUV_BW_)	5.91	1.54	5.64 to 6.17
ACC (SUV_LBW_)	3.89	0.92	3.74 to 4.05
ACC (SUV_BSA_)	5.93	1.40	5.69 to 6.17
% ID per ml (10^−3^)	8.75	2.23	8.12 to 9.39
Bone density (HU)	106.52	58.00	96.57 to 116.46
LAC (cm^−1^)	0.168	0.007	0.167 to 0.170

*ACC* activity concentration, *BW* body weight, *BSA* body surface area, *CI* confidence interval, HU Hounsfield units, *% ID* percentage of injected dose, *LAC* linear attenuation coefficient, *LBW* lean body weight, *SD* standard deviation, *SUV* standardised uptake value.

**Figure 2:**
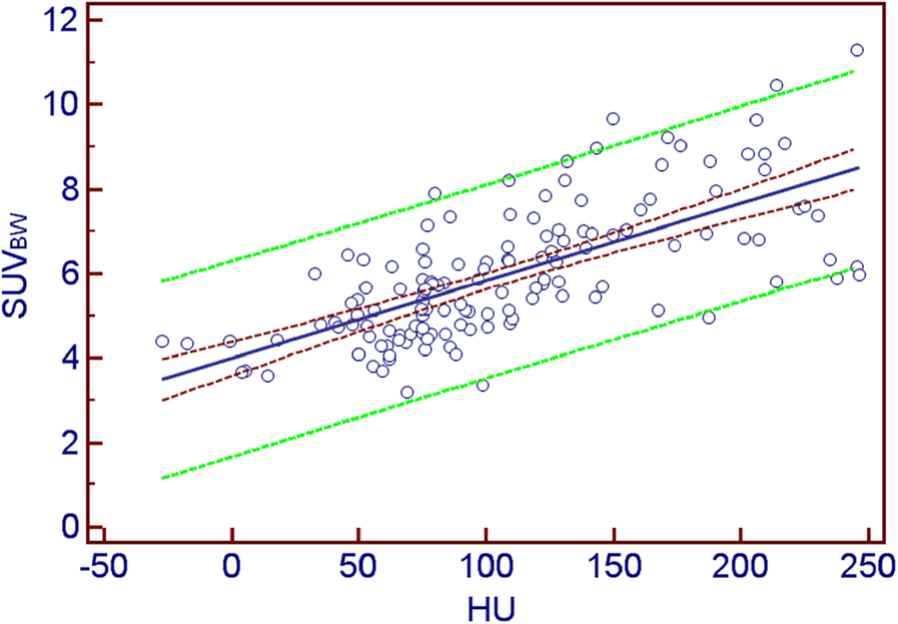
**Correlation plot between ACC and bone density.** ACC (expressed as SUV_BW_) is on the vertical axis, and bone density (HU) is on the horizontal axis. The dark red dashed curves depict the 95% confidence interval, the green dashed curves are the borders of the 95% prediction interval and the blue line shows the linear regression line fit.

Activity concentration (kBq/ml) correlated significantly with HU (*r* = 0.483, *p* < 0.0001; 95% CI, 0.341 to 0.604). ACC normalised for body mass and injected activity, SUV_BW_, showed even a stronger correlation (*z* = 2.41, *p* < 0.05) with bone density expressed in HU (*r* = 0.678, *p* < 0.0001; 95% CI, 0.575 to 0.761). SUV_LBW_ and SUV_BSA_ reported comparable statistically significant correlations (*r* = 0.648, *p* < 0.0001 and *r* = 0.637, *p* < 0.0001, respectively). Figure 2 shows the correlation plot with 95% confidence and prediction intervals on an example of SUV_BW_ and HU dependence.

**Figure 3:**
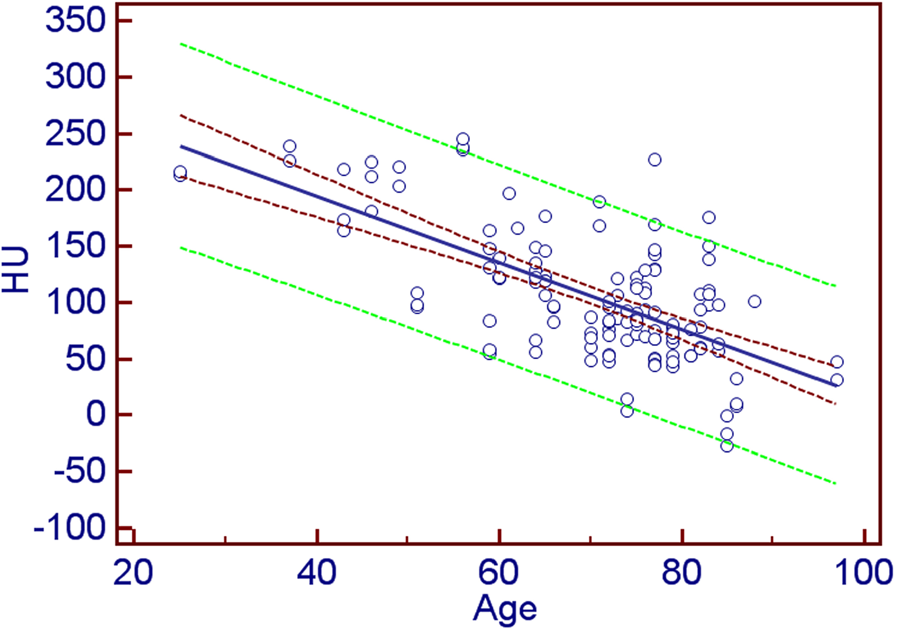
**Correlation plot between bone density and age.** Bone density (HU) is on the vertical axis, and age (years) is on the horizontal axis. The dark red dashed curves depict the 95% confidence interval, the green dashed curves are the borders of the 95% prediction interval and the blue line shows the linear regression line fit.

**Figure 4:**
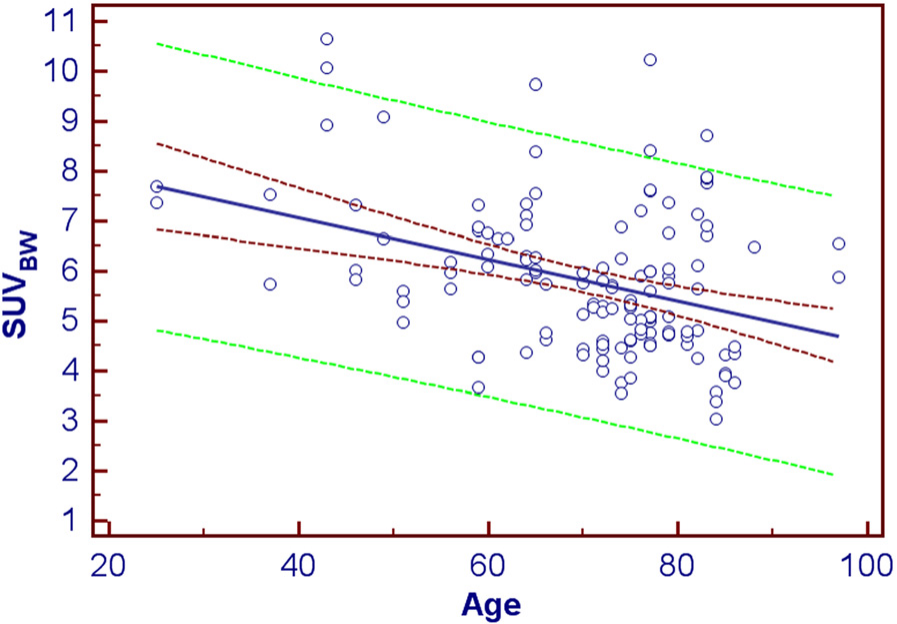
**Correlation plot between ACC and age.** ACC (expressed as SUV_BW_) is on the vertical axis and age (years) is on the horizontal axis. The dark red dashed curves depict the 95% confidence interval, the green dashed curves are the borders of the 95 % prediction interval and the blue line shows the linear regression line fit.

The correlation of absolute tracer concentration (kBq/ml) and age was also significant (*r* = −0.379, *p* < 0.0001). Significant negative correlation was observed between ACC in SUV_BW_ and age (*r* = −0.385, *p* < 0.0001; 95% CI, −0.521 to −0.229) as well as between bone density (HU) and age (*p* = −0.650, *p* < 0.0001; 95% CI, −0.739 to −0.5408) and LAC and age (*p* = −0.698, *p* < 0.0001; 95% CI, −0.776 to −0.599). Figure 3 shows the correlation plot between HU and age. Figure 4 visually describes the SUV_BW_ vs. age relationship. In the case of SUV_LBW_ and SUV_BSA_, the data showed comparable statistically significant negative correlations with age (*r* = −0.452, *p* < 0.0001 and *r* = −0.424, *p* < 0.0001, respectively). Table 2 reports on the correlations between the various measures.

**Table 2:** Correlation analyses between the measures of tracer uptake and the CT-based bone density variables

	**HU correlation ( *r *)**	**Age correlation ( *r *)**	**LAC correlation ( *r *)**
ACC (kBq/ml)	0.483	−0.379	0.422*
ACC (SUV_BW_)	0.678	−0.385	0.586
ACC (SUV_LBW_)	0.648	−0.452	0.570
ACC (SUV_BSA_)	0.637	−0.424	0.552
HU		−0.650	0.960
LAC	0.960	−0.698	

*ACC* activity concentration, *BSA* body surface area, *BW* body weight, *HU* Hounsfield unit, *LAC* linear attenuation coefficient, *LBW* lean body weight, *r*, Pearson's correlation coefficient, *SUV*, standardised uptake value. *Correlation significance for LAC vs. ACC (kBq/ml) was *p* < 0.001. All the other correlations were *p* < 0.0001.

The results collected using the two different VOI selection methods correlated significantly (*p* < 0.0001). The coefficient of determination (*R*^2^) values for HU, ACC (kBq/ml) and SUV were 0.95, 0.94 and 0.91, respectively. The mean underestimation of activity in the selected VOIs was 23.6% ± 1.3%.

### Discussion

In contrast to positron emission tomography (PET), SPECT is, to date, not routinely used for quantifying ACC in absolute terms in the clinical context. This is due to the lack of protocols correcting for the factors confounding ACC measurement by SPECT such as tissue attenuation and scatter. However, with the advent of SPECT/CT offering CT-based attenuation correction for routine clinical use, the efforts to develop truly quantitative SPECT have considerably gained in momentum. For example, Zeintl et al. [17] studied quantitative recovery of SPECT for various sphere sizes and for increasing the iteration number. In their subsequent work, they quantified the ACC of ^99m^Tc-DPD in SPECT/CT images of the bladder, compared it to ACC determined in urine samples measured in a well counter cross-calibrated against the SPECT system used and reported a mean quantitative accuracy of 1.1% ± 8.4% (95% CI, −15.4% to 17.5%) [8]. Likewise, Willowson et al. [18] studied absolute ACC for cardiac applications in phantoms using corrections for attenuation and scatter and reported a mean difference of −1% (range, −7% to 4%) for the calculated tracer activities. In their clinical validation study [7], they compared tracer activities measured using SPECT in the cardiac cavity after the injection of ^99m^Tc-labelled erythrocytes with ACC determined in blood samples and reported a mean error of about 1% (range, −6% to 5%). Such previous work shows that ACC measurements in absolute terms are feasible in a clinical setting with regular SPECT protocols.

Quantitative estimations go hand in hand with system calibration in the routine. PET systems are calibrated on a daily basis. For current SPECT routine homogeneity verifications, quality controls and peaking calibrations are performed. A regular calibration with a ^99m^Tc-filled uniform phantom as proposed in the study of Zeintl et al. [8] would be beneficial for quantitative SPECT to ensure that system volume sensitivity is stable or needs adjustments in the computations. In our experience, the system behaviour is stable over time. The sensitivity can vary between SPECT systems from different vendors and for differently sized phantoms as Seret et al. [19] have shown. They report relatively small error bars (−3.83%, 4.15% and 0.76%) for a NEMA standard phantom with a SPECT/CT Symbia system similar to those used in this study.

Building on the protocol developed by Zeintl et al., we extend their work to determine ACC in spongious bone tissue in patients referred for skeletal scintigraphy, which is one of the most common nuclear medical procedures. We compared the VOI selection using thin layers in the middle of the vertebral body and with ellipsoids, which contained most of the spongious bone in the vertebral body. The high *R*^2^ value between the two approaches shows the validity of the thin-layer VOI selection approach.

Research aimed at quantifying the uptake of diphosphonates by the skeleton was conducted in the 1970s and 1980s. Various groups [20-22] used whole body planar examinations along with blood and urine sampling to quantify tracer uptake. One-day retention of ^99m^Tc-DPD was reported by Buell et al. to be about 40% [21]. The results from these publications also served as a basis for the radiation dose report published by Weber et al. that contains dose calculations for various bone imaging agents [23].

However, owing to the problems in quantifying ACC in humans described in this study, data on the regional ACC in, for example, spongious bone is still scarce. Israel et al. used an empirical thresholding approach to quantify the concentration of ^99m^Tc-MDP 2 to 4 h after injection *in vivo* on SPECT data sets reconstructed with filtered back projection without attenuation or scatter correction and reported a mean concentration percentage of about 5 × 10^−3^ of injected activity per millilitre in the lumbar spine for normal patients [8]. Israel et al. did not report the resulting ACC in kilobecquerel per millilitre. Based on the reported injected activity ranging from 740 to 925 MBq, the ACC would be in the range of 37 to 46.25 kBq/ml. We used ^99m^Tc-DPD SPECT data which were reconstructed using OSEM with depth-dependent 3D resolution recovery as well as correction for attenuation and scatter. We also corrected the injected activity for the residual activity remaining in the syringe after the injection (8.11% ± 2.97%), minimising quantitative errors. In our data, mean tracer uptake as measured in CT- and SPECT-unremarkable spongious bone of the lumbar spine was 8.75 ± 2.23 × 10^−3^ % ID per ml and 48.15 ± 13.66 kBq/ml. The higher DPD concentration compared to MDP is consistent with the properties of these imaging agents reported by Buell et al. [21].

Zeintl et al. [8,17] simulated the behaviour of SPECT in spherical lesions using phantoms and defined correction tables for partial volume (PV). In our study, we computed the underestimation of the activity based on their PV correction methodology and obtained a mean tracer activity underestimation of 23.6% ± 1.3%. The correction tables were defined for spherical lesions having a lesion-to-background contrast ratio of 1 to 12 in the work of Zeintl et al. [8]. In order to compute an AC underestimation factor, a value interpolation between the known VOI sizes is necessary. This is not possible for VOIs of non-spherical shape which behave differently. We anticipate that the quantitative error in the vertebral body is smaller due to cross talk from the adjacent osseous tissue not only in the axial but also in the dorsal direction. To prove this assumption, a phantom simulation study needs to be conducted which is out of the scope of this paper. Partial volume correction methods based on phantoms and simulations will clearly give accurate results for phantoms and a good approximation for similarly shaped regions with homogeneous activity distribution in patients, such as for the bladder as shown in the study of Zeintl et al. [8]. In order to correct for partial volume effects in more complex structures and lesions, simulation studies adapted to their shape and size will have to be performed, ideally tailored to each individual patient. However, this is not feasible on a routine clinical basis. Different VOI selection, e.g. data maximum, or thresholding techniques even lead to a more complex behaviour which can exaggerate noise and create overshoots common in OSEM as well as Gibbs artefacts in the reconstructed volume [24].

In PET, ACC measurement is part of the clinical routine. Frost et al. [25] determined ACC 1 h after intravenous injection of [^18^F]fluoride to be 5.5 SUV. With our results, we give more insight to SPECT bone ACC, identify tracer uptake in female patients 4 h post injection and report a mean tracer uptake of 5.91 SUV for ^99m^Tc-DPD in spongious osseous tissue that is of the same magnitude as for [^18^F]fluoride uptake in PET 1 h post injection. Clearly, PET is superior to SPECT in terms of spatial resolution when today's imaging technology is considered. Nevertheless, SPECT bears the potential of technological improvement; furthermore, SPECT has the advantage of lower costs and, at least at present, better availability than PET in most countries [26].

The diphosphonates bind to the calcium-rich tissue and the mineral phase of bone hydroxyapatite [27,28]. Bone density correlates with hydroxyapatite's physical and structural density in the bones as well as with HU determined using CT [29]. Our results show, the thus expected, strong correlation of tracer activity concentration with bone density expressed in HU (*r* = 0.678).

Bone density decreases with age, in particular in women [30,31]. The significant correlation between bone density in HU and patient age is thus not unexpected. Bone density as well as age correlated significantly with normalised ACC expressed as SUV (*r* = 0.678 and *r* = −0.385) as well as with ACC (kBq/ml) (*r* = 0.483 and *r* = −0.379, respectively). The significantly stronger correlation of SUV and HU (*z* = 2.41, *p* < 0.05) compared to ACC (kBq/ml) and HU can be attributed to the reduction of the dependence of the correlations tested on administered dose as a confounding variable in this analysis.

CT measurement of HU is patient-dependent and its precision varies strongly with patient size and the body part examined. By incorporating a calibration phantom in the scanned FOV, quantitative CT (QCT) can correct for these factors [32]. In our study, we simulated QCT using attenuation maps generated during SPECT reconstruction. The results showed highly significant correlations between LAC and SUV (*r* = 0.586, *p* < 0.0001) and suggest further investigation of this type of approach for bone density measurement.

Our data paved the road towards the usage of ACC and SUV in clinical routine for diagnostic purposes. One interesting indication in this context would be to use these variables to monitor therapy of bone metastases. More specifically, they could be used as adjunct to the RECIST criteria which to date have neglected the behaviour of bone metastases on CT and magnetic resonance images for methodological reasons [33].

The significant correlation between ACC and SUV on the one hand and HU and age on the other suggests the use of quantitative SPECT to study osteoporosis or other diseases involving bone metabolism. Nevertheless, QCT would be expected to be more precise for quantification of bone loss. QCT, however, does not allow the assessment of dynamic indices of bone metabolism such as bone turnover. Cook and co-workers studied quantitative [^18^F]fluoride PET in a dynamic acquisition setting [34] and could, therefore, also calculate kinetic parameters of skeletal fluoride uptake. Zeintl et al. [35] have shown that quantitative dynamic SPECT is feasible with current-generation, commercially available equipment. SPECT/CT thus harbours the potential for the precise assessment of diseases affecting bone turnover such as osteoporosis.

Osseous ACC of ^99m^Tc-DPD reflects bone metabolism only indirectly as this variable is also influenced by tracer delivery to the osseous tissue. To allow for the calculation of the transfer constants describing bone metabolism, a measurement of the time radioactivity concentration curves in the arterial blood is necessary, which was not performed in this study. Furthermore, the binding of the polyphosphonate used by plasma proteins would have to be taken into account. The latter point is also important in view of the differences in plasma protein binding reported for the different bone-seeking ^99m^Tc-labelled agents [36]. All of these factors may be influenced by various diseases, e.g. renal insufficiency or hyperparathyroidism so that their analysis is important also in the clinical context.

A limitation of this paper is that we investigated DPD in adult women only. Therefore, the data proffered can only cautiously be generalised to men or younger subjects. All patients in whom SPECT/CT had been performed had indeterminate lesions in the lumbar region on planar scans which in most cases corresponded to degenerative disease of the spine as to be expected in the age group studied. The VOIs for measuring ACC and bone density were, however, only placed in spongious bone tissue not exhibiting focal SPECT or CT abnormalities. Therefore, the values reported herein are not affected by degenerative or malignant diseases.

## Conclusions

The SUV values determined by SPECT/CT for DPD uptake of CT- and SPECT-normal spongious bone are in the same range as those reported for [^18^F]fluoride measured using PET. The strong correlation of SUV with bone CT density underlines the physiological significance of this variable. Our data suggest the further investigation of the potential value of ACC measurement in the diagnosis of pathological conditions such as osteoporosis or in the follow-up of osseous metastases under treatment.

## Competing interests

Michal Cachovan and Joachim Hornegger have a research cooperation in the field of SPECT/CT with Siemens Healthcare, Molecular Imaging. Hans Vija works for Siemens Healthcare, Molecular Imaging. Torsten Kuwert gives lectures on behalf of Siemens Healthcare and has a research cooperation in the field of SPECT/CT.

## Authors’ contributions

MC conceived the study, participated in its design and coordination, carried out measurements, data processing and evaluation, and drafted the manuscript. AHV participated in the design of the study and evaluation and helped draft the manuscript. JH participated in the design of the study and coordination and helped draft the manuscript. TK conceived the study, participated in its design, coordination and evaluation, and drafted the manuscript. All authors read and approved the final manuscript.
